# Early number word learning: Associations with domain-general and domain-specific quantitative abilities

**DOI:** 10.3389/fpsyg.2022.1024426

**Published:** 2022-10-25

**Authors:** Meiling Yang, Junying Liang

**Affiliations:** ^1^Foreign Studies College, Northeastern University, Shenyang, China; ^2^Department of Linguistics, Zhejiang University, Hangzhou, China

**Keywords:** cardinal number knowledge, domain-general skills, domain-specific skills, family background factors, young children

## Abstract

Cardinal number knowledge-understanding “two” refers to sets of two entities-is a critical piece of knowledge that predicts later mathematics achievement. Recent studies have shown that domain-general and domain-specific skills can influence children’s cardinal number learning. However, there has not yet been research investigating the influence of domain-specific quantifier knowledge on children’s cardinal number learning. The present study aimed to investigate the influence of domain-general and domain-specific skills on Mandarin Chinese-speaking children’s cardinal number learning after controlling for a number of family background factors. Particular interest was paid to the question whether domain-specific quantifier knowledge was associated with cardinal number development. Specifically, we investigated 2–5-year-old Mandarin Chinese-speaking children’s understanding of cardinal number words as well as their general language, intelligence, approximate number system (ANS) acuity, and knowledge of quantifiers. Children’s age, gender, parental education, and family income were also assessed and used as covariates. We found that domain-general abilities, including general language and intelligence, did not account for significant additional variance of cardinal number knowledge after controlling for the aforementioned covariates. We also found that domain-specific quantifier knowledge did not account for significant additional variance of cardinal number knowledge, whereas domain-specific ANS acuity accounted for significant additional variance of cardinal number knowledge, after controlling for the aforementioned covariates. In sum, the results suggest that domain-specific numerical skills seem to be more important for children’s development of cardinal number words than the more proximal domain-general abilities such as language abilities and intelligence. The results also highlight the significance of ANS acuity on children’s cardinal number word development.

## Introduction

Cardinal number learning is a challenging but essential skill for young children before they begin formal school mathematics education. Research has shown that while children are endowed with a range of numerical knowledge and skills at an early age, it takes them a long time to develop cardinal number knowledge (Wynn, 1992; Lei, 2019). It has been found that most children aged two and half years acquire the cardinal meaning of number word *one* while all other numbers are simply considered larger than *one*. Over the next 2 or 3 years, children acquire the cardinal meaning of *two*, *three* and *four* in order. Sometime after this period, children suddenly seem to be able to generate the right cardinality for *five* and larger numbers. That is, children finally figure out how the last number word in their count list determines the numerosity of the sets. The protracted developmental trajectory of cardinal number learning indicates that cardinal number learning poses particular difficulties for children. Therefore, it seems essentially important to identify skills associated with cardinal number learning and incorporate these skills into instruction/intervention to improve cardinal number learning. There are practical reasons too for finding out about the factors associated with cardinal number development. Early mathematical skills include a range of different skills, among which early cardinal number skills are more emphasized than other abilities. Clements and Sarama (2007) emphasized that children’s development of counting and cardinality serves as the “capstone of early numerical knowledge, and the necessary building block for all further work with number and operations” (p. 467). For both practical and theoretical reasons, therefore, there is a pressing need for empirical research to identify factors associated with cardinal number learning.

Not surprisingly, much research on mathematics development in the past several decades has explored skills associated with cardinal number development. It has been found that domain-general skills such as language ability (e.g., Santos and Cordes, 2021), intelligence level (e.g., Traverso et al., 2021), and working memory capacities (e.g., Noël, 2009; Roggeman et al., 2015), can predict children’s cardinal number learning. In addition to research on domain-general predictors of cardinal number learning, there is a growing literature showing that domain-specific factors, such as counting (Aunola et al., 2004), finger skills (e.g., Pecyna et al., 2019), and subtizing skills (Paliwal and Baroody, 2020), have an influence on children’s cardinal number learning. However, the influence of domain-specific quantifier knowledge on cardinal number development has not been considered in the existing studies. Quantifiers (such as *some*, *many*) in natural language are considered as a particularly intriguing class of words for communicating quantities (Pezzelle et al., 2018). To understand the meaning of quantifiers, children need to discover that (a) quantifiers denote properties of sets rather than properties of individual objects, (b) quantifiers denote set relations (e.g., *some*) or, in certain cases, proportions of sets (e.g., *most*), and (c) *some* denotes “some,” *many* denotes “many”-that is they must discover which specific relations or proportions quantifiers denote (Barner et al., 2009). As with the case of quantifiers, in order to understand cardinal number words, children first need to discover that number words denote properties of sets. Second, they must discover that number words denote the cardinalities of sets. Finally, they must discover which specific cardinality each number word denotes. Given these similarities between cardinal number words and quantifiers, children’s knowledge of quantifiers may serve as a scaffold for learning cardinal number words. Therefore, it seems necessary to examine the influence of domain-specific quantifiers on the development of cardinal number words. Furthermore, most of the previous studies on the impact of domain-general and domain-specific skills on the development of cardinal number knowledge have focused on English and other European languages. Research on cardinal number development has shown differences in the patterns of cardinal number learning between children who speak Asian languages such as Mandarin Chinese and children who speak Indo-European languages such as English. For example, Le Corre et al. (2016) compared Mandarin Chinese-speaking children’s acquisition of the meaning of cardinal number words with that of English-speaking children. They found that Mandarin Chinese-speaking children learn the meaning of the number word for *one* 3 to 6 months later than do English-speaking children. There is also a range of evidence revealing cross-cultural variations in the impact of domain-specific skills on the learning of cardinal number words between the two languages. Yang and Wang (2022) reported that domain-specific skills, such as knowledge of quantifiers, appear to play a more important role in English than in Mandarin Chinese. Thus, these findings suggest that it is particularly important to understand the development of cardinal number words in Chinese and find out the factors that promote Chinese children’s cardinal number learning.

Given these considerations, and in conjunction with the theoretical framework that proposes three pathways for cardinal number development (LeFevre et al., 2010), the present study aimed to investigate the influence of domain-general and domain-specific skills on Mandarin Chinese-speaking children’s cardinal number learning after controlling for a number of family background factors. Particular interest was paid to the question whether domain-specific quantifier ability was associated with children’s understanding of cardinal number words. Given the available evidence showing that domain-specific numerical precursor skills appear to be more important for children’s development of cardinality as well as the concept of zero compared to domain-general abilities (e.g., Pixner et al., 2018), we hypothesized that domain-specific numerical predictors such as ANS acuity would contribute more to children’s development of cardinal words than the more proximal domain-general skills such as vocabulary and intelligence in the present study.

In the following, we first briefly present the evidence for the influence of domain-general skills, particularly vocabulary and intelligence, on the development of cardinal number words. We then summarize the evidence for the influence of domain-specific skills, particularly ANS abilities, on the development of cardinal number words. We also point out the paucity of research on the influence of domain-specific quantifiers on the development of cardinal number words. Finally, we summarize the evidence for the influence of family background factors on the development of cardinal number words.

### Domain-general skills in number word development

Vocabulary ability is one of the most frequently studied domain-general predictors of early number word development. Negen and Sarnecka (2012) found that having a larger vocabulary size helps children learn cardinal number words. Further results from Slusser et al. (2019) showed that children’s general vocabulary skills may serve as the basis for developing early number word knowledge. Previous research also reported that children with specific language impairments perform poorly on basic numeracy tasks compared to those without these problems (e.g., Cross et al., 2019).

Apart from general language abilities, there is also evidence suggesting that intelligence may be associated with early numeracy development. For instance, Traverso et al. (2021) found that general intelligence is a significant predictor of early numeracy knowledge. Chu et al. (2016) found that intelligence can predict growth in a variety of early numeracy competencies, including cardinal number abilities, numeral recognition, discrete quantity discrimination, and non-verbal calculation.

Hence, children’s vocabulary ability as well as intelligence (see above) will be used as domain-general predictors of their early cardinal number development.

### Domain-specific skills in number word development

A large body of research on the impact of domain-specific skills on early numeracy development has shown that the ability to nonverbally represent the approximate numeriosity of sets of objects, often referred to as the approximate number system (ANS) acuity, plays an important role in the development of cardinal number word knowledge. It has been found that children’s ANS acuity is a robustly predictive of their cardinal number knowledge (e.g., Hornung et al., 2014; Chu et al., 2015).

In contrast to the large number of studies that have examined the influence of ANS acuity on cardinal number development, a few, but not many, studies have explored the influence of quantifier knowledge (like *many* and *most*) on the development of cardinal number words. Barner et al. (2009) investigated the relation between cardinal number learning and quantifier comprehension in a sample of 58 English-speaking children and found that children who had a better cardinal number knowledge also had better quantifier knowledge. Furthermore, a correlation between cardinal number knowledge and quantifier knowledge in Japanese-speaking children was also observed (Barner et al., 2009). In agreement with these two studies, Dolscheid et al. (2017) also found a significant correlation between quantifier and number knowledge in German-speaking children. These findings seem to indicate that domain-specific quantifier knowledge may be a relevant predictor of children’s early understanding of the cardinality.

Hence, children’s ANS acuity and quantifier knowledge will be used as domain-specific predictors of their cardinal number development.

### Family background factors in number word development

Of course, children’s early numeracy development is also influenced by characteristics of the child and family. Many different demographic factors of early numerical development have been identified, including age, gender, parental education and family income, which are all associated with children’s numeracy development. Age has always been considered an important determining factor for cardinal number development. It has been found that children’s numeracy skills improved across years (e.g., Chu et al., 2016). In addition to age, children’s gender has also been shown to be related to children’s early numeracy development (e.g., Anders et al., 2012).

In addition to the above factors, prior studies have shown that family socioeconomic status (often operationalized as a composite measure of parental education and family income) is particularly important for developing early numeracy knowledge. For example, Anders et al. (2012) reported that parents’ educational level predicted children’s numeracy skills in the first year of preschool and their development in the next 2 years. In addition to parents’ educational background, family income has also been found to be positively related to early numeracy development. For example, Ramani and Siegler (2011) found that 4-year-old children from middle-income backgrounds had better numerical knowledge than children from low-income background.

Taken together, these prior studies suggest that child and family backgrounds have an influence on children’s numeracy development. Therefore, age, gender, parental education, and family income were controlled for and used as covariates in the present study.

### The present study

As highlighted above, children’s cardinal number development may be influenced by domain-general (i.e., general vocabulary and intelligence) and domain-specific factors (i.e., ANS acuity and quantifier knowledge). The present study aimed to examine the relative contribution of domain-general and domain-specific factors to the development of children’s cardinal number knowledge while controlling for child and family background factors. Specifically, we assessed 2–5-year-old Mandarin Chinese-speaking children’s cardinal number knowledge. In addition to an assessment of children’s cardinal number development, we assessed domain-general abilities of general vocabulary and intelligence as well as domain-specific abilities of ANS acuity and quantifier knowledge. A number of child and family background factors, including age, gender, parental education, and family income, were controlled for as covariates.

The present study extended previous research in several ways. First, the present study investigated the influence of domain-general and domain-specific factors on the learning of cardinal number words in a sample of Mandarin Chinese-speaking preschoolers. This issue has been largely overlooked in previous studies. Second, the present study is the first attempt to examine the influence of quantifier knowledge as a domain-specific numeracy skill on children’s development of cardinal number words. Third, there are only very few studies that pay specific attention to the link between domain-general and domain-specific precursors of children’s cardinal number knowledge. In this respect, the present study will enrich existing literature by providing empirical evidence on how domain-general and domain-specific skills contribute to the development of children’s cardinal number words.

## Materials and methods

### Participants

Participants were 38 preschoolers ranging in age from 2.81 to 5.75 years (*M* = 4.16 years; *SD* = 0.86 years; 19 girls). Three of these participants were 2-year-olds (1 boys, 2 girls); 14 were 3-year-olds (6 boys, 8 girls); 12 were 4-year-olds (8 boys, 4 girls); and 9 were 5-year-olds (4 boys, 5 girls). The age range was chosen in order to make our results comparable to previous research on children’s cardinal number learning (e.g., Barner et al., 2009). Demographic information such as age, gender, parental education, and family income was obtained from each participant through parent questionnaires. All participants in the study spoke Mandarin Chinese as their primary language. Parent education was recorded based on the maximum level of education reported for either parent. The range of parent education was from no formal schooling to a graduate degree. Family income was recorded based on the annual *per capita* income and grouped into three categories according to the annual *per capita* income standard by the National Bureau of Statistics of China, i.e., lower-middle level, middle level, and middle-upper level. An additional 4 children (3 girls and 1 boy, *M* = 3.00 years, *SD* = 0.20) were eliminated from the study because they failed to respond to the experimenter across the first few implementation trials in the Count list elicitation task. Children were included in the analyses for which they had completed the relevant tasks. Therefore, sample sizes were slightly reduced from the maximum sample (*N* = 38) for each set of analyses. Written consents were obtained from children’s parents and all participants received a small gift for their participation. All experimental procedures conformed with the Research Ethics Board of Zhejiang University. The study was also reviewed and approved by the Research Ethics Board of Zhejiang University.

### Measures and procedures

Children were met individually in a separate quiet room during school hours and completed all the tasks in two sessions. In the first session, children were asked to complete a total of three tasks: the Count list elicitation task, the Give-a-Number task and the Give-a-Quantifier task, in this order. In the second session, children were requested to complete the receptive vocabulary task, the ANS acuity task, and IQ testing, in this order. Each session lasted approximately 30–50 min.

### Count list elicitation task

The count list elicitation task was administrated to ensure that children knew the verbal count list for numbers at least up to 10. The experimenter began the task by asking the child questions like, “Can you count?” If a child hesitated or made an error in counting to 10, the child was encouraged to count a row of 10 identical stickers. In the case of sticker-counting, the experimenter assisted the child’s counting by saying, “one, two, three.” while pointing to one sticker at a time. If a child failed to count correctly up to 10 when counting the stickers, the child was allowed to count again up to a total of three attempts.

### Give-a-number task

The Give-a-Number task was adapted from Wynn (1992). In this task, the experimenter presented a pile of plastic cars and a puppet doll in front of the child and prompted the child to give the puppet doll a certain number of cars. The child was always requested 1 car at first. If he succeeded in giving 1 car, 2 cars would be further requested; otherwise, the child would be requested to check and fix answers. For each trial thereafter, if the child succeeded in giving N, N + 1 cars would be requested, otherwise, N-1 cars would be requested. This pattern of titration continued until the child successfully gave 5 cars. A child’s knower-level was indexed as the highest number he could give correctly upon the experimenter’s request. A child was classified as an N-knower (e.g., one-knower, two-knower, three-knower, or four-knower) only when he correctly gave N at least two out of three times but failed to give N + 1 at least two out of three times. A child was credited as CP-knower (cardinal principle knower) if he could correctly give 5 cars at least two out of three times.

### Give-a-quantifier task

Following Barner et al. (2009),we used the Give-a-Quantifier task to evaluate children’s comprehension of quantifiers: *a few*, *some*, *many*, *most*, and *all*. Stimuli consisted of a red plastic circle and three sets of plastic fruit (eight oranges, eight bananas and eight strawberries) that were presented in separate piles organized by kind. The child was first asked to name the different categories of fruits to ensure that he could distinguish them. Then the experimenter prompted the child to put a quantity of a specific category of fruit into the red circle using a quantifier (e.g., “Could you put some of the oranges into the red circle?”). Examples of how each quantifier was used were presented in Table 1. Participants were each tested with quantifiers arranged in one of the four fixed orders. The pairings of the requested quantifiers and fruit kinds were quasi-random, with the restriction that each pairing was not repeated. Each participant was tested twice with each quantifier.

**Table 1:** Examples of how quantifiers were used in the give-quantifier task.

Quantifier	Examples in English translation
*A few/shaoxu*	“Could you put a few bananas into the red circle?’”
*Some/yixie*	“Could you put some bananas into the red circle?”
*Many/xuduo*	“Could you put many bananas into the red circle?”
*Most/daduoshu*	“Could you put most of the bananas into the red circle?”
*All/suoyou*	“Could you put all of the bananas into the red circle?”

### ANS acuity task

To measure children’s ANS acuity, we administrated a non-symbolic numerical comparison task using the Panamath program similar to the one used by Halberda et al. (2008). Children were presented two spatially separately arrays of blue and yellow dots simultaneously, one to the left of the screen and one to the right, and were asked to indicate which dot array contained more dots. Each dot array was displayed for 2,000 ms followed by a blank screen that remained until the child gave a response. The experimenter pressed one of the two keys on the keyboard to indicate the child’s response. The experimenter pressed “F” if the child thought the array on the left contained more dots and pressed “J” if the child thought that the array on the right contained more dots. The number of dots in each set (blue and yellow) ranged from 4 to 15. The ratio between the arrays varied randomly among four ratio bins: 1: 2, 2: 3, 3: 4, 5: 6. We controlled for surface area by using three randomly intermixed trial type. In the first control trial, the total surface area of the dots was equated across the two boxes (total filled area trials). In the second control trial, the mean surface area of the dots was equated across the two boxes (correlated area trials). In the third control trial, the surface area of the dots within each box was inversely related to numerosity (anti-correlated area trials). The experiment started with 6 practice trials to ensure that children understand the task. Following these practices, a total of 60 test trials were presented.

### Receptive vocabulary task

We administered the Peabody Picture Vocabulary Test (PPVT-4; Dunn and Dunn, 1981) to test children’s receptive vocabulary size. Following the standard testing protocol, children were shown a plate with four pictures and were asked to indicate which picture best corresponded to the stimulus word spoken by the experimenter. Children received several practice trials before the formal test to ensure that they understand the task. Once the formal test began, each participant had to answer eight consecutive questions correctly in order to meet the basal requirement. Testing ended when the child reached the ceiling, making six errors within eight consecutive responses. Raw scores were used in all the analyses.

### IQ testing

The Chinese Binet-Simon Scale-Revised was used to measure children’s intelligence levels (Wu, 1982). This test consists of 51 items that measure a wide range of cognitive capacities including verbal discrimination, abstract thinking, and reasoning ability, etc. Testing ended when a child failed to pass five test items consecutively.

## Results

### Count list elicitation task

The count list elicitation task assessed children’s knowledge of the verbal counting system. Our analysis revealed that all the children tested were able to recite the count list at least to 10 in sequence.

### Give-a-number task

Based on their performance in the Give-a-Number task, children were classified into the following number-knower levels: one-knowers (*n* = 2), two-knowers (*n* = 7), three-knowers (*n* = 5), four-knowers (*n* = 2), and CP-knowers (*n* = 22). See Table 2 for the age range of each of the knower-level.

**Table 2:** Ages of children by number-knower level in the give-a-number task.

Number-knower level	*N*	Age (years)	
		Mean (*SD*)	Range
One-knowers	2	3.43 (0.02)	3.41–3.45
Two-knowers	7	3.18 (0.32)	2.81–3.62
Three-knowers	5	3.47 (0.05)	3.40–3.54
Four-knowers	2	4.08 (0.07)	4.03–4.14
CP-knowers	22	4.67 (0.70)	2.99–5.75
Total	38	4.14 (0.85)	2.81–5.75

### Give-a-quantifier task

**Figure 1:**
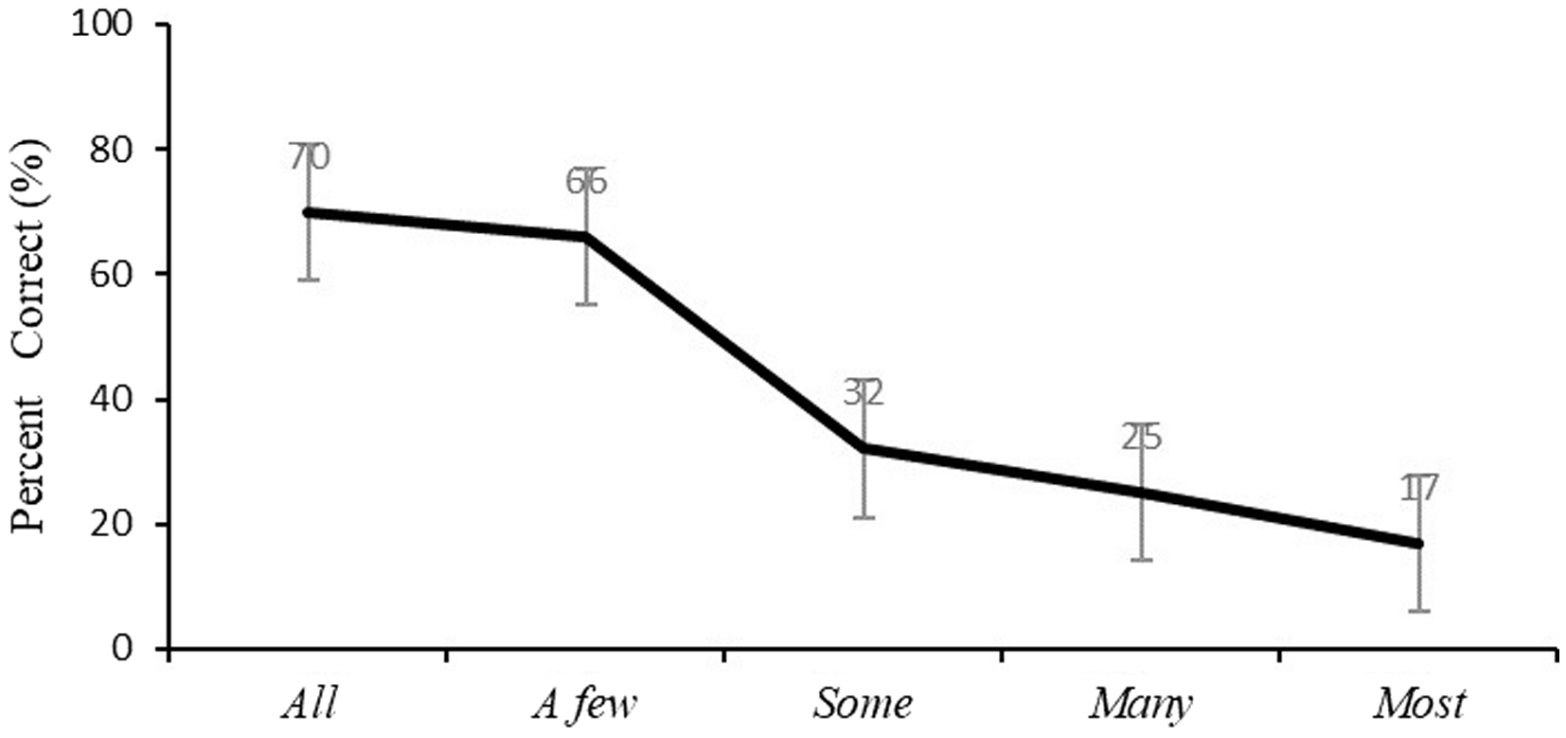
Children’s average percent correct for each quantifier.

Following Barner et al. (2009), children’s correct response for each quantifier is defined according to the judgment of adult speakers of Mandarin Chinese (*n* = 23). For each quantifier, adult participants were asked to determine the possible number of objects among 8 that might be asked on two trials. The criteria shown in Table 3 were determined by the broadest range of possible number of objects reported by adults upon each quantifier. Children’s comprehension of quantifiers was analyzed in terms of the number of correct responses that they provided over two trials for each quantifier (resulting in scores of 0, 1, or 2). Figure 1 presents children’s average percent correct for each quantifier.

**Table 3:** Definitions of “correct” (adult-like) responses for each quantifier.

Quantifier	Correct responses
*A few*	1–3
*Some*	2–4
*Many*	4–7
*Most*	5–7
*All*	8

Total number of objects: eight.

### ANS acuity task

Children’s performance on the ANS acuity task was analyzed in terms of accuracy (percent correct), because accuracy has been thought to provide the most reliable and valid measure of humans’ number acuity (e.g., Clayton et al., 2015). On average, children responded correctly on 74% of the trials, showing above-chance performance, *t*(36) = 12.00, *p* < 0.001, eta = 0.03. Kruskal-Wallis test was conducted to examine the differences in the ANS accuracy according to area control conditions. No significant differences were found among the total filled area trials, correlated area trials and anti-correlated area trials, χ^2^(2) = 5.90, *p* > 0.05. We therefore collapsed over, the total filled area trials, correlated area trials and anti-correlated area trials in further analyses. Kruskal-Wallis test with factor number ratio (1: 2, 2: 3, 3: 4, and 4: 5) showed that children’s accuracy decreased with increasing number ratio (i.e., 83%, 77%, 68%, and 67%), χ^2^(3) = 23.45, *p* < 0.001, eta = 0.14.

### Receptive vocabulary task

Children’s receptive vocabulary scores were kept in raw form, ranging from 14 to 103 (*M* = 46.60, *SD* = 24.93). Raw score was used because of its sensitivity in detecting small differences in the study of young children’s cognitive development (Fletcher et al., 1991; Sullivan et al., 2014).

### IQ testing

Children’s intelligence test scores ranged from 68 to 108, with an average IQ of 86.97 (*SD* = 10.05).

### Multiple regression analyses for number word knowledge

Table 4 summarizes the descriptive statistics of performance scores of each age group on domain-general and domain-specific tasks. Table 5 presents the intercorrelations among all the variables. As shown in Table 5, cardinal number knowledge correlated with age, receptive vocabulary, and ANS acuity at the significance level of *p* < 0.001. IQ was also correlated with cardinal number knowledge. In contrast, gender, parental education, family income, and quantifier knowledge did not correlate with cardinal number word knowledge, and consequently would not be included as covariates in further analyses.

**Table 4:** Summary of descriptive statistics of performance scores on domain-general and domain-specific tasks for each age group.

	Two-year-old	Three-year-old	Four-year-old	Five-year-old
	M (SD)	M (SD)	M (SD)	M (SD)
Domain-general factors				
Vocabulary (raw scores)	26.67 (14.18)	28.86 (12.65)	50.25 (15.06)	76.00 (23.72)
Intelligence (standard scores)	84.67 (4.62)	85.46 (10.67)	88.33 (10.69)	88.11 (10.60)
Domain-specific factors				
ANS (percent correct)	67.22 (9.77)	65.26 (9.59)	76.94 (8.90)	85.37 (9.99)
Quantifiers (raw scores)	4.67 (1.15)	3.93 (1.14)	4.17 (1.59)	4.44 (1.67)

**Table 5:** Intercorrelations among the study variables.

Variable	1	2	3	4	5	6	7	8	9
1. Number word knowledge	–								
2. Gender (0 = girl, 1 = boy)	0.05	–							
3. Age (years)	0.73**	0.08	–						
4. Parental education	−0.07	−0.17	−0.34	–					
5. Family income	−0.05	0.03	−0.18	0.31	–				
6. Receptive vocabulary	0.65**	0.10	0.75**	−0.21	−0.17	–			
7. IQ	0.37*	0.16	0.23	−0.13	−0.06	0.47**	–		
8. ANS acuity	0.73**	0.10	0.72**	−0.22	−0.28	0.62**	0.40*	–	
9. Quantifier knowledge	0.21	−0.13	−0.01	0.07	0.26	0.13	0.003	−0.04	–

* *p* < 0.05;

** *p* < 0.01.

A multiple regression model predicting number word knowledge by age, receptive vocabulary, IQ, and ANS acuity was conducted. Age was found to be the only significant predictor of children’s number word knowledge (*p* = 0.007). We then examined whether children’s domain-general skills (receptive vocabulary, IQ) and domain-specific skills (ANS acuity) contributed to individual differences in number word knowledge above and beyond age separately. The multiple regression analyses revealed that when controlling for age, children’s domain-general skills (receptive vocabulary, IQ) did not explain a significant amount of variance in their number word knowledge, *F*(3,30) = 2.84, *p* = 0.07, f2 = 0.19, whereas children’s domain-specific skills (ANS acuity) explained a significant amount of variance in their number word acquisition *F*(2,31) = 4.40, *p* = 0.04, f2 = 0.14. All statistical analyses were performed in R (version 4.0.5).

## Discussion

Over the past decade, an increasing body of research has examined how domain-general and domain-specific abilities contribute to children’s development of numeracy skills. However, most of the studies have primarily focused on elementary school students and mainly considered the development of early numeracy skills such as mental number line, number identification, while neglecting the development of cardinal number words. Moreover, the influence of domain-specific knowledge of quantifiers on the development of cardinal number words has also received little attention. Also, the majority of research on cardinal number development and its relation to domain-general and domain-specific skills has been conducted with children who speak English and European languages. To fill these gaps, the present study examined the influence of domain-general skills and domain-specific abilities on Mandarin Chinese-speaking children’s development of cardinal number words. Specifically, we aimed at investigating possible differential prediction of domain-general abilities such as language skills and intelligence as well as domain-specific abilities such as ANS acuity and quantifier knowledge on children’s understanding of cardinal number words. The present study yielded two important findings. First, domain-specific ANS acuity, but not domain-specific quantifier knowledge was a significant predictor of children’s knowledge of cardinal number words after controlling for covariates such as age. Second, although domain-general skills, including vocabulary and intelligence, were found to be associated with children’s cardinal number development, they were no longer significant predictors of cardinal number knowledge after controlling for covariates such as age.

As mentioned earlier, we were particularly interested in the influence of domain-specific quantifier knowledge on children’s cardinal number development. However, contrary to our expectations, we found that domain-specific quantifier knowledge did not predicted cardinal number development after controlling for covariates such as age. This seems to be in contrast to the previous findings showing that children’s comprehension of quantifiers (like *all*, *many*) correlates with their cardinal number knowledge (e.g., Barner et al., 2009). However, a closer look at these studies suggests that there are good reasons to believe that Mandarin Chinese children’s knowledge of quantifiers may not be relevant to learning cardinal number words. Recent research on the relationship between cardinal number learning and quantifier comprehension has shown that the link between them varies across languages (Barner et al., 2009). Specifically, it has been found that there is a strong correlation between cardinal number learning and quantifier comprehension among English-speaking children, while the correlation between them is much weaker among Japanese-speaking children. Similarly, Yang and Wang (2022) provide evidence that there is no significant correlation between cardinal number learning and quantifier comprehension in Mandarin Chinese-speaking children. Taken together, these findings suggest that while quantifier knowledge may provide a scaffold for cardinal number learning in children who speak languages such as English, it may play a lesser role in children who speak classifier languages such as Japanese and Mandarin Chinese. One possible explanation for this is that languages differ in the availability of grammatical features that connect cardinal number learning with quantifier learning. In contrast to the consistent syntactic overlap between cardinal number words and quantifiers in English, quantifiers are used in variable and different syntactic frameworks in many respects from the use of number words in Mandarin Chinese (Le Corre et al., 2016). For example, in Mandarin Chinese, both number words and quantifiers can be used to modify nouns, but whenever a number word is used to modify a noun, it generally has to be used in conjunction with a classifier such as “ge,” functioning as individuation. However, when a quantifier is used to modify a noun, the use of a classifier is optional (Zhang, 2007). The varying syntactic patterns in which number words and quantifiers appear in Mandarin Chinese may potentially put Mandarin Chinese-speaking children at a disadvantage in terms of discovering cardinal number meanings from quantifiers. However, we also acknowledge that the small number of participants included in the present study may make it difficult to detect correlations between cardinal number words and quantifiers. This asks for future studies evaluating the association between cardinal number abilities and quantifier knowledge in a larger sample and in children from different linguistic backgrounds.

With regard to the influence of ANS acuity, our findings are in line with earlier findings (e.g., van Marle et al., 2016) arguing for the importance of ANS acuity for the development of children’s early numeracy abilities. Moreover, our findings fit into those of Mussolin et al. (2012), suggesting that the precision in numerosity discrimination appears to play a more critical role in the development of children’s early numeracy abilities than later mathematics achievement. The association between ANS acuity and formal mathematics skills may imply that children’s approximate number sense may underlie formal mathematics learning and thus may help children develop formal math skills. However, it is also possible that the approximate number sense itself is refined by formal mathematical learning, in which case the refinement of the approximate number sense may be less important and useful. Our study shows that number discrimination accuracy, measured before the onset of formal education, predicts the development of cardinal number words even after controlling for age. This finding is hard to reconcile with the idea that approximate number sense is a reflection of the quality of school mathematics instruction a child has received. Instead, our finding may be more supportive of this idea that ANS actually underlies mathematics learning. Support for this view provided by recent investigation showing that the association between ANS acuity and mathematical abilities progressively weakens with age (Mussolin et al., 2012).

Apart from investigating the influence of domain-specific precursors of cardinal number development, we were interested in the influence of domain-general skills of vocabulary and intelligence on cardinal number development. We found that general vocabulary abilities and intelligence were associated with children’s cardinal number development. The finding that cardinal number knowledge was significantly correlated to domain-general abilities such as intelligence and receptive vocabulary appears to demonstrate that basic domain-general abilities are important abilities involved in comprehending and learning exact number word meanings. This finding is consistent with extant findings in the literature that general intelligence is tied to and predict many aspects of mathematics (Roth et al., 2015). It is also consistent with past results showing that having a larger receptive vocabulary helps children learn the meaning of exact number words (Praet et al., 2013). In addition, our finding also suggests that children’s age is a consistent predictor of children’s cardinal number development. This finding echoes with many previous findings showing that children’s cardinal number knowledge increased with age (e.g., Rousselle and Vossius, 2021). The age-related change is possibly related to the growing experiences with counting in everyday play and activities (Gallistel and Gelman, 1992). It is also possible that children’s growth in cardinal number knowledge may rely on age-related increases in other cognitive capacities, such as ANS acuity or subtizing skills (Benoit et al., 2004).

Finally, at a broad level, our findings suggest that domain-specific skills seem to be more strongly related to children’s cardinal number development than domain-general skills. This finding joins others in demonstrating that relative to domain-general skills, domain-specific skills tend to be more strongly related to preschool numeracy competences (Chu et al., 2015, 2016). It may be that domain-specific numeracy skills are particularly important at some early stages when children need to build abstract knowledge of natural number concepts. Our finding is again consistent with previous studies showing that while domain-general factors have been found to contribute to the development of children’s early numeracy skills, the effects are often indirect mediated by domain-specific numerical competence (Passolunghi and Lanfranchi, 2012). It also fits the view that language learning is a dynamic multicausal process involving the interactions of multiple systems in which domain-general ability is just one of the many factors that influence the learning (Herdina and Jessner, 2002).

In sum, the present study extends our understanding of the factors that are involved in the development of cardinal number knowledge, but several questions remain open. First, although a substantial number of studies have found that ANS acuity facilitates symbolic mathematics development, we are far from understanding the mechanisms that underlie this relationship (see Szkudlarek and Brannon, 2017), possible mechanisms of this relationship. Second, domain-specific quantifier skills seem to be less related to cardinal number learning, at least in the present sample of Mandarin Chinese-speaking children. However, the explanation for this finding remains speculative.

## Conclusion

The present study examined how domain-general skills such as intelligence and receptive vocabulary and domain-specific quantitative abilities including quantifier knowledge and ANS acuity relate to the learning of cardinal number words. The results showed that after controlling for age, children’s domain-specific skills, particularly ANS acuity explained a significant amount of variance in their number word knowledge, whereas domain-general skills including IQ and receptive vocabulary did not explain additional variance beyond age. This finding highlights the importance of applying domain-specific ANS knowledge when learning number words and adds more evidence to the association between ANS acuity and cardinal number learning.

## Data availability statement

The raw data supporting the conclusions of this article will be made available by the authors, without undue reservation.

## Ethics statement

The studies involving human participants were reviewed and approved by Research Ethics Board of Zhejiang University. Written informed consent to participate in this study was provided by the participants’ legal guardian/next of kin.

## Author contributions

MY and JL conceived and designed the research, and wrote the manuscript. MY performed the research and analyzed the data. All authors contributed to the article and approved the submitted version.

## Conflict of interest

The authors declare that the research was conducted in the absence of any commercial or financial relationships that could be construed as a potential conflict of interest.

## Publisher’s note

All claims expressed in this article are solely those of the authors and do not necessarily represent those of their affiliated organizations, or those of the publisher, the editors and the reviewers. Any product that may be evaluated in this article, or claim that may be made by its manufacturer, is not guaranteed or endorsed by the publisher.
